# Six weeks open-label oral ketamine for patients with treatment-resistant depression, post-traumatic stress disorder, or obsessive-compulsive disorder

**DOI:** 10.1177/02698811251344710

**Published:** 2025-06-05

**Authors:** Ben Beaglehole, Paul Glue, Shona Neehoff, Shabah Shadli, Neil McNaughton, Bridget Kimber, Chrissie Muirhead, Aroha de Bie, Rachel Day-Brown, Natalie J Hughes-Medlicott

**Affiliations:** 1 Department of Psychological Medicine, University of Otago, Christchurch, New Zealand; 2 Department of Psychological Medicine, University of Otago, Dunedin, New Zealand; 3 Department of Psychology, University of Otago, Dunedin, New Zealand; 4 School of Psychology, Charles Sturt University, Bathurst, NSW, Australia; 5 School of Pharmacy, University of Otago, Dunedin, New Zealand

**Keywords:** Ketamine, maintenance treatment, oral, TRD, PTSD, OCD, depression

## Abstract

**Background:**

We previously completed a double-blind randomised crossover study assessing intramuscular ketamine for treatment-resistant depression (TR-D), post-traumatic stress disorder (TR-PTSD) and obsessive-compulsive disorder (TR-OCD). Here, we report an extension study to explore the ongoing benefits and tolerability of maintenance oral ketamine.

**Method:**

All participants from the original study were eligible to receive a 6-week open-label course of oral ketamine once-thrice weekly. Racemic ketamine for injection was diluted in orange juice and sipped over 30–60 min. Dose amount and frequency were adjusted individually to maximise benefits and tolerability. Effectiveness was assessed by disorder-specific scales. Side effects and tolerability were assessed using reported adverse events and scales for dissociation and urinary/bladder symptoms.

**Results:**

Seventeen participants with TR-D, 18 participants with TR-PTSD and 8 participants with TR-OCD commenced oral ketamine. Nine participants with TR-D, 16 participants with TR-PTSD and 5 participants with TR-OCD completed all 6 weeks of dosing. Ketamine dose increased over time from 1–1.5 mg/kg to 1.5–2.5 mg/kg, with a dosing frequency of 1–3 times/week, with an average total dose rising from 1.9 to 3.0 mg/kg/week over the first 3 weeks. Symptom rating scores for TR-D, TR-PTSD and TR-OCD were low at week 1 of oral dosing (compared to scores at entry into the original study) and remained low throughout the six-week course of oral ketamine. Oral ketamine was well tolerated with minimal side effects.

**Conclusion:**

The 6-week extension of oral ketamine appeared to sustain improvements for TR-D, TR-PTSD and TR-OCD. Oral ketamine was well tolerated and offers an alternative option for patients, researchers and clinicians.

## Introduction

The evidence base for ketamine treatment of psychiatric disorders is rapidly growing. It is now well-established that there are short-term benefits from single or repeated ketamine doses for treatment-resistant depression (TR-D; McIntyre et al., 2021). Other early-phase studies also report the benefits of ketamine treatment for post-traumatic stress disorder (PTSD), obsessive-compulsive disorder (OCD), anxiety disorders, eating disorders and substance use disorders (Johnston et al., 2024).

There are challenges that prevent the routine use of ketamine by clinicians. These include high rates of relapse that follow single doses or short courses of ketamine treatment (aan het Rot et al., 2010) and concern about side effects, including dissociation, cystitis and sedation, which require in-clinic ketamine administration and monitoring (aan het Rot et al., 2010; Kraus et al., 2017). The use of oral ketamine partially addresses these concerns. Oral formulations are more tolerable with fewer side effects (Kumar et al., 2024). Oral ketamine is easier to administer and is therefore more amenable to longer courses with the potential to reinforce early improvements or provide maintenance treatment (Andrade, 2023). In addition, there are equity advantages for oral ketamine due to avoiding the time, cost and resources associated with parenteral administration (Andrade, 2023; Beaglehole et al., 2023).

Systematic reviews evaluating oral ketamine for depression report that oral ketamine is well tolerated and associated with clinical improvements (although only a small number of randomised studies were identified; Meshkat et al., 2023). We are unaware of trials that have assessed oral ketamine for TR-PTSD or TR-OCD. Given the benefits we report with intramuscular (IM) dosing (Beaglehole et al., 2024a, 2024b), this area requires evaluation.

This study reports data from an open-label maintenance extension to an earlier double-blind, crossover, randomised controlled trial (RCT) evaluating ketamine compared to IM fentanyl (psychoactive control) for TR-D, TR-PTSD and TR-OCD. The principal finding from the RCT phase of the study was the short-term benefits of IM ketamine compared to IM fentanyl for all three cohorts. All participants were offered a 6-week open-label extension utilising oral ketamine subsequent to the RCT. The purpose of this phase of the study was to explore any ongoing benefits and tolerability of oral ketamine during the 6-week extension period.

## Methods

The study was registered with the Australian and New Zealand Clinical Trial Registry (ACTRN12619000311156). The authors assert that all procedures contributing to this work comply with the ethical standards of the relevant national and institutional committees on human experimentation and with the Helsinki Declaration of 1975, as revised in 2013. All procedures were approved by the Central Health and Disability Ethics Committee (19/CEN/21 approval date 17.4.2019). All participants gave written informed consent. The Strengthening the Reporting of Observational Studies in Epidemiology checklist (von Elm et al., 2007) was followed to guide the reporting of the study and is reported in the Supplemental material.

This research is part of a wider study that recruited patients with TR-D, TR-PTSD, TR-OCD and Spider Phobia in separate cohorts to evaluate the effects of ketamine on EEG biomarkers linked to trait neuroticism and clinical outcomes. The first phase of the study was a randomised double-blind crossover study comparing single doses of IM racemic ketamine 0.5 mg/kg, 1.0 mg/kg and fentanyl 50 µg (psychoactive control). The data from that phase are reported elsewhere (Glue et al., 2024; Beaglehole et al., 2024a, 2024b).

This article describes the second phase of the study, an optional open-label 6-week extension of variable dose oral racemic ketamine. Oral ketamine was not offered to participants in the spider phobia cohort. Liquid ketamine for injection (Ketalar^®^) was diluted with 50 ml of orange juice (or similar) to reduce its bitter taste and was sipped over 30–60 min to minimise dissociation. The starting dose was generally 1mg/kg based on previous experience treating patients with oral ketamine in Dunedin. Subsequent doses were individualised through discussion with overseeing clinicians to optimise target symptoms, tolerability and convenience. If there was inadequate response to 1 mg/kg, the dose could be increased to 1.5 and then 2 mg/kg (and higher in selected circumstances). If the response was of inadequate duration, frequency could increase from weekly to twice weekly and occasionally thrice weekly. The variable dose regime was operationalised to ensure consistency between centres. The duration of oral treatment was 6 weeks, following which participants were discharged to their usual healthcare providers.

Treatment and data collection occurred in the Departments of Psychological Medicine at two locations (Dunedin and Christchurch, New Zealand). The symptom scales used in the original RCT phase of the study were administered weekly prior to the first dosing session of the week and at 60 min post-dose (only the pre-dose scores are reported). These were the Hospital Anxiety Depression Scale subscales for anxiety (HADS-A) and depression (HADS-D; Zigmond and Snaith, 1983; depression cohort), the Impact of Event Scale–Revised (IESR; Weiss and Marmar, 1997; PTSD cohort) and the Yale-Brown Obsessive-Compulsive Scale (Y-BOCS; Goodman et al., 1989; OCD cohort). For each of these scales, higher scores are associated with greater symptomatology with cut-points applied at entry to the first phase of the study to ensure significant symptoms were present.

Tolerability and safety assessments included the presence of adverse events (AEs) that were recorded throughout the study. The Clinician-Administered Dissociative States Scale (CADSS; Bremner et al., 1998) was used to measure dissociative side effects of ketamine 30 min after dosing. Higher scores indicate greater dissociative experiences with a maximum score of 32. Popova et al. (2019) report dissociation to be present with a CADSS score of >4 so we also report the number of participants in each disorder group whose score was >4 at any time during oral dosing (Popova et al., 2019). Bladder symptoms were monitored using the Bladder Pain/Interstitial Cystitis Symptom Score (BPIC-SS; Humphrey et al., 2012) administered prior to the first dosing session of each week. Higher scores indicate greater symptomology, with a cut-score of 18 being suggested for Bladder Pain Syndrome (Humphrey et al., 2012). Systolic and diastolic blood pressure recordings were undertaken pre-dose and 30 min post-dose for each dosing session.

**Figure 1.**
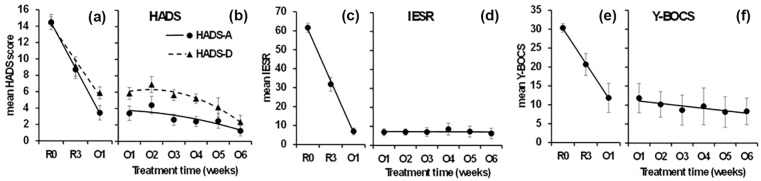
Mean (SE) symptom rating scores before and during 6 weeks of oral maintenance ketamine dosing. (Panels a and b) HADS-A and -D scores; (Panels c and d) IESR scores; (Panels e and f): Y-BOCS scores. R0 = RCT baseline; R3 = RCT week 3 (7-day time point); O1–O6 = weeks of oral treatment. Curves represent the significant trends in the data, except for panels d and f where the fitted straight lines are not significant. (a) HADS-A and HADS-D scores show a large and steady decrease in scores between R0 and O1. (b) HADS-A and HADS-D share a nonlinear decreasing trend over O1–O6. The differences between their trend curves are not significant. (c) IESR is similar to but more extreme than HADS. (d) IESR does not change significantly over O1–O6. (e and f) Y-BOCS trends are the same as IESR.

**Figure 2.**
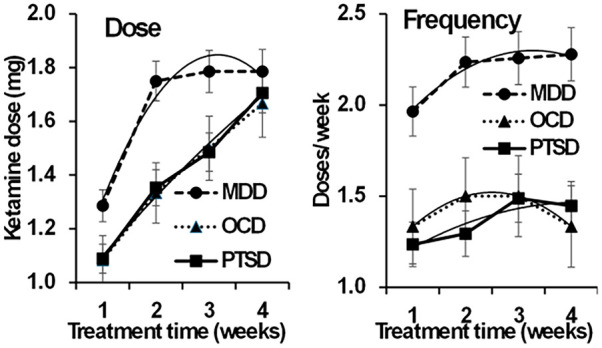
Mean (SE) ketamine dose and dose frequency by study week. Note that significance is often common across the groups with no group interaction. Triangle: OCD; Circle: MDD; Square: PTSD. Dashed lines represent polynomial trends for individual groups.

Data were extracted from case record forms and, for CADSS, BPIC-SS and blood pressure, submitted to mixed measures Analysis of Variance (ANOVA) with diagnosis as a group factor and time as the repeated measure. Occasional missing values were interpolated. For the ketamine dose (Figure 2) there were large numbers of values missing for weeks 5 and 6 and so these were eliminated from the analysis of dose but not from the analysis of clinical measures (Figure 1). For dose frequency, there was one value missing for each of the three participants. In all cases, the adjacent values were the same as each other and so this value was used to replace the missing value. With CADSS, there was one value missing from each of the two participants. The adjacent values (and the values for many other participants) were 0 and so this was used to replace the missing values. For systolic and diastolic blood pressure and for BPIC-SS, one value was missing from each of the same three participants. Replacement values were based on the values of participants with a similar pattern of adjacent values. For the IESR one value was missing from one participant on week 6 and replaced with 0 based on the trend of previous scores. Clinical measures were analysed in separate repeated measures ANOVAs for each diagnosis (HADS for TR-D; IESR for PTSD; Y-BOCS for OCD).

For both the mixed and repeated measures ANOVAs, effects across time were decomposed routinely by Statistical Package for the Social Sociences (SPSS) within the ANOVA as orthogonal polynomial contrasts (Rosenthal, 1985). For the same calculations in R and other systems, Google ‘orthogonal polynomial contrasts’. These are descriptive and independent and can be summed to recover the original means (McNaughton, 1993). Critically, with 1 numerator *df*, they are not affected by sphericity with each component having its own error term. For the same reason, because they have only 1 *df*, they do not require post hoc testing of the differences among the means to which they are fitted, and so they do not require correction for multiple post hoc comparisons. As the analysis was exploratory, with no control over the final sample size, no adjustment for the multiplicity of polynomial comparisons was deemed necessary. Summary statistics (means and standard error of the means (SEMs)) were calculated and weekly mean values for the 6-week treatment period were plotted for each patient group (TRD, TR-PTSD and TR-OCD). Where polynomial components are significant, these (or post hoc components) are plotted as curves; otherwise, standard errors are provided for individual points. Separate ANOVAs were carried out for the 6 weeks of oral testing (O1–O6) and for three sets of readings prior to oral ketamine receipt: RCT baseline (R0); RCT week 3 at 1 week after the last dosing (R3); oral week 1 (O1), which is prior to the first oral dosing.

## Results

Twenty-five participants with TR-D completed the initial RCT comparing IM ketamine and IM fentanyl. Of these participants, 17 elected to continue to the oral treatment phase and 9 received ketamine in all optional weeks. Thirty-three participants with TR-PTSD were reported in the initial RCT phase of the study, but nine of these participants had a primary diagnosis of depression, and their oral data are included in the depression cohort. Of the 24 participants with primary TR-PTSD, 18 continued with oral ketamine, and 16 participants completed all 6 weeks. Ten participants with TR-OCD completed the initial RCT, eight participants chose to continue with oral ketamine and five participants completed 6 weeks of dosing. The most common reasons for not continuing into the oral extension phase were either logistical or due to a lack of perceived benefit from the initial RCT. Most patients who did not complete 6 weeks left earlier due to scheduling difficulties or due to COVID-19 lockdowns.

The mean age of the TR-D cohort who received oral ketamine was 34 years (SD 11.7). Fifty-nine percent of this group were female. For the TR-PTSD oral ketamine cohort, the mean age was 36 years (SD: 9.6) and 89% were female. The mean age of the TR-OCD oral ketamine cohort was 35 years (SD: 3.7) and 63% were female.

The mean R0 HADS anxiety and depression sub-scale scores (prior to entering the initial RCT) for participants with TR-D were 14.5 (SEM: 0.78) and 14.7 (SEM: 0.53). These had reduced to 3.9 (SEM: 0.89) and 6.2 (SEM: 0.68) respectively by week one of the oral extension phase (O1) and remained lowered during oral dosing (Figure 1(a) and (b)). The HADS anxiety and depression sub-scale scores were 2.3 (SEM: 0.67) and 4.1 (SEM: 0.73) at O6.

As can be seen in Figure 1(a), scores decreased steadily for both HADS subscales between R0 and O1 (the linear trend was significant, time (linear) *F*(1, 13) = 130.885, *p* < 0.0001; with no deviation of R3 from the straight line, time (quadratic) *F*(1, 13) = 0.584, NS). The apparent difference between the scales was not reliable (the linear trend of the difference between the scales was not significant, scale × time (linear × linear) *F*(1, 13) = 3.674, *p* = 0.078). As can be seen in Figure 1(b), there was a tendency for scores to increase at O2 (time (quadratic) *F*(1, 13) = 5.740, *p* = 0.032) and then show an increasing downward trend (time (linear) *F*(1, 13) = 14.718, *p* = 0.002). There appeared to be a steady separation and then convergence of the two measures – with HADS-A becoming progressively lower than HADS-D and then HADS-D catching up but this was not reliable (the *U*-shape of the difference in scale scores did not achieve *p* < 0.05, scale × time (quadratic) *F*(1, 13) = 4.102, *p* = 0.064). Later values in both cases are likely to have been affected by a floor effect.

The mean R0 IESR for participants with TR-PTSD was 61.7 (SEM: 2.26). This had reduced to 6.7 (SEM: 1.78) by O1 and remained lowered throughout oral dosing (Figure 1(c) and (d)). The mean IESR at week 6 was 6.6 (SEM: 2.79). There was a steady change over R0, R3 and O1 (Figure 1(c); time (linear) *F*(1, 16) = 430.257, *p* < 0.0001; with no deviation of R3 from the linear trend time (quadratic) *F*(1, 16) = 0.502, NS). There were no significant changes over O1–O6 (all *F*(1, 16) < 2.6, all *p* > 0.1), and the straight line shown in Figure 1(d) has a slope of ~0.5%. The lack of change is likely a floor effect (c.f. HADS-A).

The mean R0 Y-BOCS for participants with TR-OCD was 30.4 (SEM: 1.32). This had reduced to 10.5 (SEM: 3.13) by O1 and remained lowered throughout oral dosing (Figure 1(e) and (f)). The Y-BOCS at week 6 was 9.0 (SEM: 3.33). As can be seen in Figure 1(a), scores decreased steadily between R0 and O1 (time (linear) *F*(1, 5) = 19.995, *p* = 0.007; with no deviation of R3 from the straight line, time (quadratic) *F*(1, 5) = 0.019, NS). There were no significant changes over O1–O6 (all *F*(1, 5) < 3.3, all *p* > 0.1), and the straight line shown in Figure 1(d) has a slope of ~5%.

Figure 2 reports the mean ketamine dose and mean weekly dosing frequency provided to each cohort over the first 4 weeks (to include all available participants) of the extension phase. The mean dose was 1.3 mg/kg (SEM: 0.06) during week 1 for TR-D, 1.1 mg/kg (SEM: 0.05) during week 1 for TR-PTSD, and 1.1 mg/kg (SEM: 0.08) during week 1 for TR-OCD. Mean ketamine doses increased during treatment for all cohorts with the exception of a small reduction from weeks 5 to 6 for TR-D. At week 6, the mean ketamine doses for TR-D, TR-PTSD and TR-OCD were 2.0 mg/kg (SEM: 0.08), 1.8 mg/kg (SEM: 0.06) and 2.0 mg/kg (SEM: 0.00) respectively. Over the 4 weeks where data for all participants is available (Figure 2, Dose), TR-D showed generally higher values (diagnosis, *F*(2, 34) = 5.708, *p* = 0.007) with a rapid initial increase that then tapered off to values that were reached more slowly by the other two groups (the difference between the *U*-shape of their functions was significant, diagnosis × time (quadratic) *F*(2, 34) = 5.292, *p* = 0.01; see dashed line for MDD).

The mean weekly dosing frequency for TR-D was 2.0 × weekly (SEM: 0.12) at week 1 and 2.2 × weekly (SEM: 0.04) at week 6. Ten participants with TR-D received thrice-weekly dosing during some part of the oral-dosing period. The mean dosing frequency for participants with TR-PTSD was 1.2 × weekly (SEM: 0.10) at week 1 which increased to 1.53 × weekly (SEM: 0.13) by week 6. For TR-OCD, the mean dosing frequency was 1.4 × weekly (SEM: 0.18) at week 1, reached a peak of 1.7 × weekly (SEM: 0.29) at week 5 and was 1.4 × weekly (SEM: 0.19) at week 6. Over the 4 weeks where data for all participants is available (Figure 2, Frequency), TR-D showed consistently higher values (diagnosis, *F*(2, 34) = 12.602, *p* < 0.001; diagnosis × time (all components) *F*(2, 34) < 2.6, all *p* > 0.09). All three diagnoses showed a similar nonlinear trend (dashed line, time (linear) *F*(1, 34) = 3.876, *p* = 0.057 NS; time (quadratic) *F*(1, 34) = 7.629, *p* = 0.0090. Dose changes were prompted by reporting of residual symptoms or insufficient duration of response. We did not experience requests for higher or more frequent dosing when symptom control was achieved, although potential participants were excluded from study entry if they had current or past 6-month substance use disorders.

**Figure 3.**
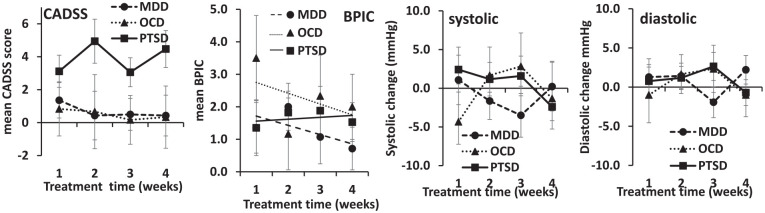
Mean (SE) CADSS, BPIC, systolic blood pressure and diastolic blood pressure (at 30 min) scores by study week. Black triangle: TRD; Open circle: PTSD; Grey square: OCD.

Mean CADSS scores at 30 min post-dosing are reported for each cohort during each week of dosing in Figure 3 (CADSS). Figure 3 (BPIC) also reports the mean BPIC scores for each week. The highest mean CADSS scores were present in the PTSD cohort (diagnosis *F*(2, 34) = 2.898, *p* = 0.069 NS) but mean scores were generally low (<6) for each patient group during all weeks of dosing and no effect of time or interaction of diagnosis with time was significant (all *F* < 2, all *p* > 0.15). When the threshold of CADSS > 4 was applied, there were three participants with TRD (18%), seven with TR-PTSD (39%) and zero with TR-OCD who met this threshold. The CADSS score range is from 0 to 92, so the reported mean scores are indicative of minimal dissociation overall. The BPIC scores were also low; peak mean BPIC scores were 2.3 (SEM: 0.71) for TR-D (week 2), 2.1 (SEM: 0.68) for TR-PTSD (week 2) and 3.1 (SEM: 2.17) for TR-OCD (week 1). The BPIC scale has a cut-off of 12 for Bladder Pain Syndrome, so these scores are also indicative of minimal urinary bladder symptoms. Scores tended to decrease with time (time (linear) *F*(1, 34) = 4.314, *p* = 0.045) but the apparent differences in rate between the diagnoses (see linear trend lines in the figure) were not reliable (diagnosis × time (linear) *F*(2, 34) = 2.434, *p* = 0.103 NS). Change in systolic and diastolic blood pressure from pre-dose to 30-min post-dose by study week is also shown in Figure 3. Minimal change in blood pressure was present for all dosing weeks and patient cohorts (all systolic *F* < 2, all *p* > 0.15; all diastolic *F* < 2, all *p* > 0.15).

Oral ketamine was generally well tolerated. The most frequent side effects reported were lightheadedness (present in 14% of dosing sessions), mild dissociation (12% of dosing sessions), nausea (2% of dosing sessions) and headaches (2% of dosing sessions). The majority of patients reported no side effects (TRD 41%–63%, PTSD 65%–77%, OCD 75%–100% of treatment sessions). No serious AEs occurred.

## Discussion

In this article, we report data from an open-label extension study that provided a 6-week course of oral ketamine for TR-D, TR-PTSD and TR-OCD. Oral ketamine was well tolerated with minimal side effects. Symptom rating scores were low at week 1 of oral dosing (compared to scores at entry into the original study) and remained low during the treatment period.

The 6-week oral ketamine course followed the initial RCT in which participants received two IM ketamine doses. Not all participants chose to continue with oral dosing (due to logistical reasons and lack of perceived benefit). In this context, symptoms of TR-D, TR-PTSD and TR-OCD were low at the time of oral ketamine commencing. Therefore, oral ketamine appeared to maintain the benefits provided during the initial RCT phase of the study and prevent symptom worsening. Whether the oral ketamine regime would also treat symptomatic TR-D, TR-PTSD and TR-OCD was not able to be confirmed in this study, although the existing evidence base evaluating oral ketamine for TR-D (Meshkat et al., 2023) provides some level of support for therapeutic as well as maintenance benefits.

The majority of ketamine studies are single-dose or short-term parenteral studies with few studies evaluating longer ketamine courses and safety data (Short et al., 2018). Unlike parenteral ketamine, oral ketamine is more typically provided for longer courses over a number of weeks. Consequently, our findings add to the existing literature reporting benefits from repeated doses of oral ketamine for TR-D. We are unaware of previous studies that evaluated oral ketamine for TR-PTSD and TR-OCD. Our findings suggest there are benefits of oral ketamine for these diagnoses.

TR-D scores fell during the oral treatment course, whereas TR-PTSD and TR-OCD scores were stable during the treatment period, possibly exhibiting a floor effect. If oral ketamine were ineffective, we would expect to see increasing symptom scores over time. The TR-D cohort tended to receive higher ketamine doses and were dosed more frequently than the other cohorts. The majority of participants with TR-PTSD and TR-OCD were treated in Christchurch, while the majority of participants with TR-D were treated in Dunedin. It is possible that practice differences between centres may account for some of the dosing differences. However, higher and more frequent doses may also be required to adequately treat TR-D. Given the small numbers in each cohort, we suggest that further work exploring the optimal dosing and frequency of oral ketamine would be beneficial.

The tolerability and safety of oral ketamine were assessed through monitoring of AEs, dissociative symptoms, bladder symptoms and blood pressure. The absence of concerning findings suggests that oral ketamine is well tolerated over a 6-week period. However, given the longer-term course of TR-D, TR-PTSD and TR-OCD, there remain unanswered questions about the optimal duration of ketamine treatment for these disorders, whether maintenance ketamine should be provided, and the long-term tolerability and safety profile of ketamine.

In our view, a shift away from studies that evaluate the efficacy of parenteral ketamine is desirable. The principal advantage of parenteral ketamine is rapid responsiveness. However, there are major issues with preserving blinding in parenteral ketamine RCTs (Muthukumaraswamy et al., 2021). As the short-term effectiveness of ketamine is now established, a more useful question is: can short-term benefits be maintained (Beaglehole et al., 2024c)? Furthermore, delivery of parenteral ketamine requires specialist clinical settings that are not readily available to typical mental health service consumers. We argue that greater use of oral ketamine is more equitable and provides a treatment pathway to consumers who cannot afford or access parenteral ketamine (Beaglehole et al., 2023). On balance, we regard greater accessibility of ketamine for the treatment of depression to be desirable. However, we recognise that ketamine is a recreational substance that can be misused, although this is less likely when ketamine is taken orally, given its slower absorption. A downside to greater ketamine accessibility is the potential for diversion to recreational use, followed by more frequent ketamine-related health problems such as ketamine cystitis. We did not experience requests for higher or more frequent dosing when symptom control was achieved although participants were selected without current or past 6-months substance use disorders.

### Limitations

This was an open-label maintenance extension to a double-blind randomised crossover study. The number of participants from each disorder group is relatively small. The oral extension was optional and it is likely that participants who perceived benefits from the RCT phase of the study were more likely to participate with expectations of positive effect. Given the high rates of relapse within 30 days following single or repeated ketamine doses (aan het Rot et al., 2010; Kraus et al., 2017), it is unlikely that the maintenance of positive benefits we observed related to carryover effects from the RCT phase. However, this possibility cannot be completely discarded, particularly since oral ketamine was provided in the positive therapeutic milieu provided by a clinical trial setting. In addition, participants and clinicians worked together to optimise the dose and frequency of oral ketamine but there was no control intervention and rating scale completion was overseen by the clinicians who provided treatment.

In conclusion, we provide further support for the benefits of oral ketamine for mood and anxiety disorders. Symptom scores for TR-D, TR-PTSD and TR-OCD remained low throughout an open-label ketamine 6-week extension study. Oral ketamine was well tolerated and appears to offer an accessible alternative for clinicians and researchers to consider.

## Supplemental Material

sj-docx-1-jop-10.1177_02698811251344710 – Supplemental material for Six weeks open-label oral ketamine for patients with treatment-resistant depression, post-traumatic stress disorder, or obsessive-compulsive disorder Supplemental material, sj-docx-1-jop-10.1177_02698811251344710 for Six weeks open-label oral ketamine for patients with treatment-resistant depression, post-traumatic stress disorder, or obsessive-compulsive disorder by Ben Beaglehole, Paul Glue, Shona Neehoff, Shabah Shadli, Neil McNaughton, Bridget Kimber, Chrissie Muirhead, Aroha de Bie, Rachel Day-Brown and Natalie J Hughes-Medlicott in Journal of Psychopharmacology
